# Fractional Anisotropy Alterations in Key White Matter Pathways Associated with Cognitive Performance Assessed by MoCA

**DOI:** 10.3390/neurolint17100154

**Published:** 2025-09-25

**Authors:** Nauris Zdanovskis, Kalvis Kaļva, Ardis Platkājis, Andrejs Kostiks, Kristīne Šneidere, Guntis Karelis, Ainārs Stepens

**Affiliations:** 1 Department of Radiology, Riga Stradins University, Dzirciema iela 16, LV-1007 Riga, Latvia; 2 Department of Radiology, Riga East University Hospital, Hipokrata iela 2, LV-1038 Riga, Latvia; 3 Institute of Public Health, Riga Stradins University, Dzirciema iela 16, LV-1007 Riga, Latvia; 4 Department of Neurology and Neurosurgery, Riga East University Hospital, Hipokrata iela 2, LV-1038 Riga, Latvia

**Keywords:** diffusion weighted MRI, DWI, DTI, fractional anisotropy, neuroradiology, white matter integrity

## Abstract

**Objectives:** This study investigated fractional anisotropy (FA) differences within key white matter tracts across patient groups stratified by Montreal Cognitive Assessment (MoCA) scores, aiming to evaluate FA’s potential as a biomarker for cognitive impairment. **Methods:** Seventy participants (aged 57–96 years) were categorized into high (HP, MoCA ≥ 26), moderate (MP, MoCA 18–25), and low (LP, MoCA < 18) cognitive performance groups. Diffusion Tensor Imaging (DTI) was used to obtain FA values in corticospinal tracts, superior longitudinal fasciculus, inferior fronto-occipital fasciculus, and cingulum. Statistical analyses included ANOVA and post-hoc tests. **Results:** Significant differences in FA values and normative percentiles were observed across cognitive groups in several tracts. Notably, the MP group exhibited significantly higher FA values in the Left Superior Longitudinal Fasciculus—Arcuate (mean FA 0.329 vs. LP 0.306, *p* = 0.033) and Right Superior Longitudinal Fasciculus—Arcuate (mean FA 0.329 vs. LP 0.306, *p* = 0.009), Left Inferior Fronto-Occipital Fasciculus (mean FA 0.308 vs. LP 0.283, *p* = 0.021), and Right Inferior Fronto-Occipital Fasciculus (mean FA 0.289 vs. LP 0.266, *p* = 0.017) compared to the LP group. **Conclusions:** Our findings reveal significant FA alterations across MoCA-defined cognitive groups, with moderate impairment showing higher FA than low performance. This suggests FA may reflect complex microstructural changes in early cognitive decline. While our modest sample size, particularly in the low-performance group, limits definitive conclusions, these results highlight the need for larger, multimodal studies to validate FA’s role as a sensitive, albeit complex, biomarker for cognitive impairment.

## 1. Introduction

Cognitive impairment, ranging from mild cognitive impairment (MCI) to severe cognitive impairment (SCI) and dementia, poses significant challenges to individuals and healthcare systems globally. As populations age, the prevalence of these conditions is expected to rise further, making early detection and a nuanced understanding of the underlying neural mechanisms crucial for effective intervention and management strategies [1]. 

Findings in cortical thickness [2,3,4,5] and volumetric analyses [6,7,8,9] have provided valuable insights into structural changes associated with cognitive decline. However, these measures alone are insufficient to fully explain the complexities of cognitive impairment and, in some cases, are detectable only after clinical symptoms appear. This is where DTI and fractional anisotropy (FA) could come into play, offering a more nuanced understanding of white matter integrity and an early biomarker for cognitive decline [10,11,12,13]. Fractional anisotropy (FA) is a measure derived from DTI that quantifies the directional coherence of water diffusion in white matter tracts. There are several studies that indicate FA changes in young adults, aging [10,14], cognitive impairment, dementia [15], and other neurodegenerative conditions [16]. At present, the relationship between FA values and cognitive performance, particularly across different levels of cognitive impairment, remains underexplored. Recent findings indicate that FA decline is more closely linked to tau than Aβ, and that lower FA levels precede and predict the progression of white matter hyperintensities, highlighting microstructural degeneration as an earlier marker of disease than macrostructural changes [17]. Furthermore, emerging evidence shows that some interventions, such as carotid endarterectomy, can improve cognition and preserve hippocampal FA, underscoring the clinical relevance of FA as both a biomarker and potential treatment target [18]. Complementary large—scale work has also shown that white matter free water (FW)—a measure reflecting neuroinflammation and atrophy—explains the strongest variance in cognitive decline, particularly in limbic tracts such as the fornix and cingulum, and interacts with APOE ε4 status, hippocampal atrophy, and amyloid positivity to accelerate memory decline. Together, these findings suggest that integrating FA and FW-corrected metrics may provide a more sensitive and multimodal framework for detecting early microstructural vulnerability in aging and Alzheimer’s disease [19].

The novelty of this study lies in its combined focus on normative-adjusted FA metrics and MoCA-based three-level stratification to interrogate potential non-linear white matter alterations across cognitive performance levels. Rather than assuming a monotonic decline in FA with worsening cognition, we explicitly test whether moderate impairment can show distinct FA patterns relative to both high and low performers. By harmonizing FA values to age- and sex-specific normative data and examining tract-specific effects in SLF/IFOF and related pathways, our approach aims to clarify whether FA captures early, complex microstructural changes relevant to cognitive decline. Accordingly, our primary objective is to determine whether FA differs systematically across MoCA-defined groups and to assess the interpretability of any non-linear trends in the context of early cognitive impairment.

## 2. Materials and Methods

This section describes the participant demographics, MRI acquisition and processing, tract-based FA extraction, and statistical analyses used to examine the relationship between white matter integrity and cognitive performance. The novelty of the study is the use of normative, age- and sex-adjusted FA percentiles combined with MoCA-based stratification into three cognitive groups, allowing us to test for non-linear patterns of white matter alterations across different levels of cognitive impairment.

### 2.1. Participants

In total, 70 participants, aged 57 to 96 years, were recruited for this study. Participants were divided into three groups based on their Montreal Cognitive Assessment (MoCA [20] scores:

- High cognitive performance (HP) group (participants with MoCA scores ≥ 26), median age 72.0 years, mean age of 72.6 years (SD = 5.2), age range is from 61 to 83 years; median MoCA score is 27.0, mean score of 27.3 (SD = 1.1), MoCA scores range from 26 to 30.
- Moderate cognitive performance group (MP) group (participants with MoCA ≥ 18 and ≤25), median age 74.0 years, mean age of 72.7 years (SD = 6.8), age range is from 57 to 85 years; median MoCA score is 23.0, mean score of 22.7 (SD = 2.3), MoCA scores range from 18 to 25.
- Low cognitive performance group (LP) group (participants with MoCA ≤ 17), median age is 79.5 years, mean age of 75.9 years (SD = 11.1), age range is from 62 to 96 years; median MoCA score is 10.0, mean score of 9.7 (SD = 4.4), MoCA scores range from 4 to 16.

Descriptives for each group can be seen in Table 1.

Age differences among the three cognitive performance groups (HP, MP, and LP) were analysed using a Kruskal–Wallis test. Although the MP and LP groups were slightly older, the analysis revealed no statistically significant age differences across the three groups (Kruskal-Wallis H = 1.07, *p* = 0.586). This finding indicates that age was not a significant factor differentiating the groups.

A chi-square test of independence was then conducted to examine the relationship between cognitive group (HP, MP, LP) and gender (female, male). The association was not statistically significant, *X*^2^ (2, *n* = 70) = 5.42, *p* = 0.067.

Further analyses on the effects of age and gender on the results are discussed in the Results section.

Cognitive functions were assessed by a board-certified neurologist specializing in cognitive impairment diagnostics. Participants in our study were referred to the neurologist based on recommendations from their primary care physicians or due to their own subjective complaints of cognitive impairment.

Participants were excluded from this study if they had clinically significant neurological or psychiatric disorders (such as a history of tumours, severe strokes, vascular malformations, major depression, Parkinson’s disease, schizophrenic disorders, bipolar disorders, maniacal states, etc.), as well as a history of drug or alcohol abuse.

No other clinically significant abnormalities were detected on the MRI scans of patients enrolled in this study. None of the participants had signs of cerebral amyloid angiopathy, more than four microbleeds, intra-/extra-axial tumors, vascular malformations, or other neurodegenerative diseases. According to available clinical records, none of the participants had uncontrolled hypertension, diabetes mellitus, or clinically verified depression.

All participants were university graduates with at least 16 years of education.

### 2.2. MRI Acquisition

All participants underwent magnetic resonance imaging (MRI) using a 3.0 Tesla scanner (General Electric (GE), Boston, MA, USA) with sequences, including 3D T1 SPGR, 3D FLAIR, High-resolution T2 hippocampal sequence, Diffusion tensor imaging (DTI) (with 32 directions, 2 b values (0 and 1000 s/mm^2^), and susceptibility weighted imaging (SWI).

### 2.3. DTI Processing and Fractional Anisotropy Calculation

The DTI assessment was conducted based on Icometrix DTI icobrain tbi report values, which evaluate fractional anisotropy in Left Corticospinal Tract (LCST), Right Corticospinal Tract (RCST), Left Superior Longitudinal—Arcuate Fasciculus (LSLF-A), Right Superior Longitudinal—Arcuate Fasciculus (RSLF-A), Left Inferior Fronto-Occipital Fasciculus (LIFO), Right Inferior Fronto-Occipital Fasciculus (RIFO), Left Cingulum (LCin), Right Cingulum (RCin).

**Figure 1:**
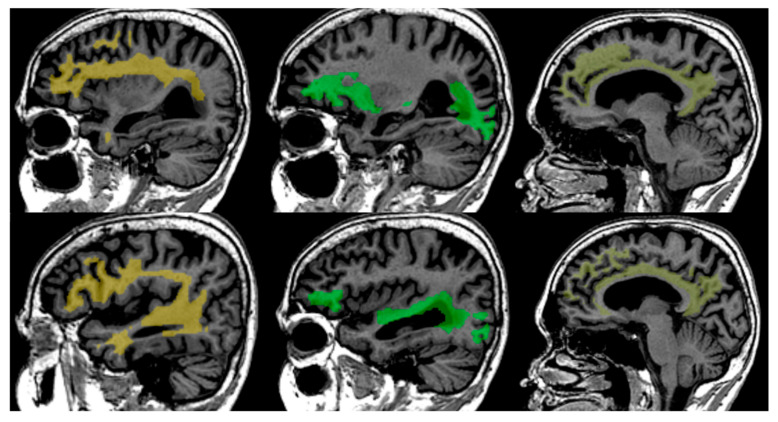
Tract segmentation using TractSeg from T1 images from left to right—Superior Longitudinal—Arcuate Fasciculus (SLF-A, bright yellow colour), Inferior Fronto-Occipital Fasciculus (IFO, green colour), Cingulum (Cin, light yellow colour).

Performed steps for processing included eddy current correction with affine registration, the use of Tractseg (see Figure 1) for creating a binary tract mask [21], and then computing the mean FA from iteratively reweighted linear least squares in the tract mask [22]. Further, FA maps and distributions are calculated and extracted from the region of interest. After preprocessing, the resulting FA data are standardised, harmonised, and compared to age- and gender-normative data obtained from a healthy population database that consists of 918 MR studies from 788 unique patients aged from 18 to 86 years, acquired on different scanners and equally distributed over different age groups [23,24,25].

In our study, we focused on fractional anisotropy (FA) values in specific white matter tracts and the normative percentile, which takes into account age and gender-based norms.

### 2.4. Statistical Analysis

Statistical analysis was performed using JASP version 0.19.0 [26]. The analysis included descriptive statistics, a chi-square test, a Kruskal-Wallis test, and Dunn’s post hoc analysis with Bonferroni corrections.

Descriptive statistics were calculated to summarize general variables, identify differences between groups, and detect correlations. The chi-square test assessed associations between categorical variables. The Kruskal-Wallis test evaluated statistically significant differences among the three groups. If significant differences were found, Dunn’s post hoc test was applied with Bonferroni correction.

### 2.5. Ethical Considerations

The study was conducted in accordance with the ethical standards of the institutional review board and the Helsinki Declaration. All participants provided written informed consent prior to participation. Participants were assured of their right to withdraw from the study at any time without consequence. Data were anonymized to ensure confidentiality and privacy.

## 3. Results

The absolute FA values and FA normative percentiles based (standardised, harmonised age- and gender-normative data) were extracted from white matter tracts, including:

- Left Corticospinal Tract (LCST)
- Right Corticospinal Tract (RCST)
- Left Superior Longitudinal—Arcuate Fasciculus (LSLF-A)
- Right Superior Longitudinal—Arcuate Fasciculus (RSLF-A)
- Left Inferior Fronto-Occipital Fasciculus (LIFO)
- Right Inferior Fronto-Occipital Fasciculus (RIFO)
- Left Cingulum (LCin)
- Right Cingulum (RCin)

Descriptive Statistics for each tract are shown in Table 2.

Statistically significant differences between cognitive performance groups were found in:

- Left Superior Longitudinal Fasciculus—Arcuate (LSLF-A)
- Right Superior Longitudinal Fasciculus—Arcuate (RSLF-A)
- Left Inferior Fronto-Occipital Fasciculus (LIFO)
- Right Inferior Fronto-Occipital Fasciculus (RIFO)

### 3.1. Statistical Analysis of Left Superior Longitudinal—Arcuate Fasciculus (LSLF-A) FA and Normative Percentile

Fractional anisotropy results in the high performance (HP) group (*n* = 21) had a mean LSLF-A FA of 0.310 (SD = 0.035, SE = 0.008), the low performance (LP) group (*n* = 14) had a mean LSLF-A FA of 0.306 (SD = 0.024, SE = 0.007), and the moderate performance (MP) group (*n* = 35) had a mean LSLF-A FA of 0.329 (SD = 0.031, SE = 0.005).

Fractional anisotropy normative percentile values HP group (*n* = 21) had a mean normative percentile of 0.536 (SD = 0.392, SE = 0.085), the LP group (*n* = 14) had a mean normative percentile of 0.524 (SD = 0.347, SE = 0.093), and the MP group (*n* = 35) had a mean normative percentile of 0.731 (SD = 0.281, SE = 0.047).

**Figure 2:**
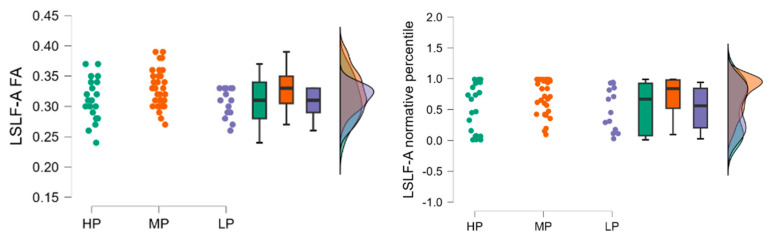
Distribution of LSLF-A FA (**left**) and normative percentile (**right**) values across the HP, MP, and LP groups.

The raincloud plot (Figure 2) visualizes the distribution of LSLF-A FA values across the HP, MP, and LP groups, highlighting the MP group showing higher median FA values compared to the HP and LP groups.

The Kruskal-Wallis test was conducted to evaluate the differences in LSLF-A FA and normative percentile values among the three groups. The test indicated a statistically significant difference in LSLF-A FA values between the groups (H = 6.235, *p* = 0.044) and in LSLF-A normative percentile values (H = 6.304, *p* = 0.043). Dunn’s post hoc tests were performed to identify specific group differences, and the results are summarized in Table 3.

For LSLF-A FA values, there was a significant difference between the MP and LP groups (z = 2.135, *p* = 0.033), indicating that the MP group had significantly higher LSLF-A FA values compared to the LP group. No significant differences were found between the HP and MP groups (z = −1.922, *p* = 0.055) or the HP and LP groups (z = 0.419, *p* = 0.675).

For LSLF-A normative percentile values, there was a significant difference between the MP and LP groups (z = 2.127, *p* = 0.033), indicating that the MP group had significantly higher normative percentile values compared to the LP group. No significant differences were found between the HP and MP groups (z = −1.957, *p* = 0.050) or the HP and LP groups (z = 0.384, *p* = 0.701).

### 3.2. ANCOVA and Covariate Controls

To further clarify these group differences, an ANCOVA was performed for both raw FA and FA normative percentiles, treating Group (HP, MP, LP) as a fixed factor while Age (covariate) and Gender (fixed factor) were controlled.

For raw LSLF-A FA values, the group remained significant (F (2, 65) = 3.67, *p* = 0.031, partial η^2^ = 0.10, Cohen’s f ≈ 0.34), indicating a moderate effect even after adjusting for age and gender.

For LSLF-A normative percentiles, the group effect was not significant, F (2, 65) = 2.93, *p* = 0.060, partial η^2^ = 0.083, Cohen’s f ≈ 0.30, reflecting a borderline difference once typical demographic variance is factored in.

In both models, gender was not a significant predictor (*p* > 0.10), whereas age was strongly significant for raw FA (*p* < 0.001) but not for normative percentiles (*p* ≈ 0.08), consistent with the notion that normative scores already incorporate age-related adjustments.

### 3.3. Statistical Analysis of Right Superior Longitudinal—Arcuate Fasciculus (RSLF-A) FA and Normative Percentile

Fractional anisotropy results in high performance (HP) group (*n* = 21) had a mean RSLF-A FA of 0.313 (SD = 0.036, SE = 0.008), the low performance (LP) group (*n* = 14) had a mean RSLF-A FA of 0.306 (SD = 0.021, SE = 0.006), and the moderate performance (MP) group (*n* = 35) had a mean RSLF-A FA of 0.329 (SD = 0.022, SE = 0.004).

**Figure 3:**
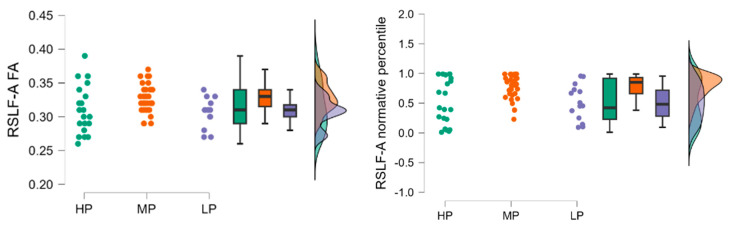
Distribution of RSLF-A FA (**left**) and normative percentile (**right**) values across the HP, MP, and LP groups.

Fractional anisotropy normative percentile results had a mean normative percentile of 0.526 (SD = 0.383, SE = 0.084), the LP group (*n* = 14) had a mean normative percentile of 0.514 (SD = 0.299, SE = 0.080), and the MP group (*n* = 35) had a mean normative percentile of 0.778 (SD = 0.198, SE = 0.033), see Figure 3.

The raincloud plots visualize the distribution of RSLF-A FA and RSLF-A normative percentile values across the HP, MP, and LP groups. The plots highlight the differences in values among the groups, with the MP group showing higher median values compared to the HP and LP groups.

The Kruskal-Wallis test was conducted to evaluate the differences in RSLF-A FA and normative percentile values among the three groups. The test indicated a statistically significant difference in RSLF-A FA values between the groups (H = 8.792, *p* = 0.012) and in RSLF-A normative percentile values (H = 8.271, *p* = 0.016).

Dunn’s post hoc tests were performed to identify specific group differences. For RSLF-A FA values, there was a significant difference between the MP and LP groups (z = 2.614, *p* = 0.009), indicating that the MP group had significantly higher RSLF-A FA values compared to the LP group. No significant differences were found between the HP and MP groups (z = −2.179, *p* = 0.088) or the HP and LP groups (z = 0.652, *p* = 0.514). 

For RSLF-A normative percentile FA values. For RSLF-A normative FA, a significant difference was observed between the MP and LP groups (z = 2.528, *p* = 0.011) and HP and MP groups (z = −2.124, *p* = 0.0t34). No significant differences were found between the HP and MP groups (z = −2.00, *p* = 0.052) or between the HP and LP groups (z = 0.75, *p* = 0.455). Dunn’s post hoc tests were performed to identify specific group differences, and the results are summarized in Table 4.

### 3.4. ANCOVA and Covariate Controls

To further account for demographic variables, an ANCOVA was performed with RSLF-A FA as the dependent variable, Group as a fixed factor, and Age (covariate), along with Gender (fixed factor) as controls.

The ANCOVA revealed a significant main effect of group on raw RSLF-A FA (F (2, 65) = 4.06, *p* = 0.022, partial η^2^ = 0.11, Cohen’s f ≈ 0.34). In contrast, the ANCOVA using normative percentiles produced a borderline group effect, F (2, 65) = 2.93, *p* = 0.060, partial η^2^ = 0.083, Cohen’s f ≈ 0.30. Notably, age was a significant predictor for raw FA but not for normative percentiles, and gender did not significantly contribute to either model.

### 3.5. Statistical Analysis of Left Inferior Fronto–Occipital Fasciculus (LIFO) FA and Normative Percentile

**Figure 4:**
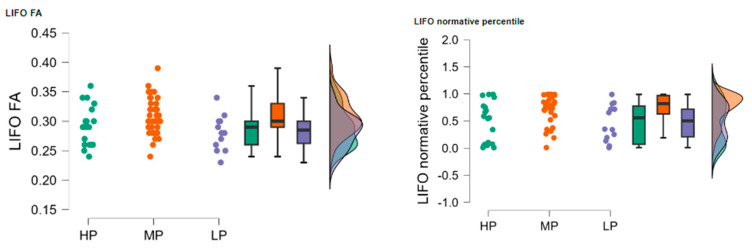
Distribution of LIFO FA (**left**) and normative percentile (**right**) values across the HP, MP, and LP groups.

The high performance (HP) group (*n* = 21) had a mean LIFO FA of 0.289 (SD = 0.034, SE = 0.007), the low performance (LP) group (*n* = 14) had a mean LIFO FA of 0.283 (SD = 0.029, SE = 0.008), and the moderate performance (MP) group (*n* = 35) had a mean LIFO FA of 0.308 (SD = 0.032, SE = 0.005). The HP group (*n* = 21) had a mean normative percentile of 0.487 (SD = 0.386, SE = 0.084), the LP group (*n* = 14) had a mean normative percentile of 0.484 (SD = 0.329, SE = 0.088), and the MP group (*n* = 35) had a mean normative percentile of 0.738 (SD = 0.274, SE = 0.046), see Figure 4.

The Kruskal-Wallis test was conducted to evaluate the differences in LIFO FA and normative percentile values among the three groups. The test indicated a statistically significant difference in LIFO FA values between the groups (H = 7.719, *p* = 0.021) and in LIFO normative percentile values (H = 9.174, *p* = 0.010).

Dunn’s post hoc tests were performed to identify specific group differences. For LIFO FA values, there was a significant difference between the HP and MP groups (z = −2.209, *p* = 0.027) and between the MP and LP groups (z = 2.315, *p* = 0.021). No significant differences were found between the HP and LP groups (z = 0.355, *p* = 0.723).

For LIFO normative percentile values, there was a significant difference between the HP and MP groups (z = −2.528, *p* = 0.011) and between the MP and LP groups (z = 2.404, *p* = 0.016). No significant differences were found between the HP and LP groups (z = 0.182, *p* = 0.856). Dunn’s post hoc tests were performed to identify specific group differences, and the results are summarized in Table 5.

### 3.6. ANCOVA and Covariate Controls

To further evaluate these group differences while accounting for demographic factors, an ANCOVA was performed with LIFO FA as the dependent variable, Group (HP, MP, LP) as a fixed factor, and Age (covariate) plus Gender (fixed factor) in the model. The group effect remained significant (F (2, 65) = 4.31, *p* = 0.018, partial η^2^ = 0.12, Cohen’s f ≈ 0.35), indicating that differences in LIFO FA persist even after controlling for age and gender. In contrast, an ANCOVA on LIFO normative percentiles showed a more modest group effect (F (2, 65) = 2.90, *p* = 0.061, partial η^2^ = 0.08, Cohen’s f ≈ 0.30), Although this borderline result suggests that once typical age- and sex-related variations are factored into the normative calculation, the differences between cognitive groups become less pronounced, the effect size still indicates a potentially meaningful distinction.

In both models, Age was a strong predictor of raw LIFO FA but not of LIFO normative percentiles, consistent with the built-in demographic adjustments in the normative approach. Gender did not significantly affect LIFO FA in either model.

### 3.7. Statistical Analysis of Right Inferior Fronto–Occipital Fasciculus (RIFO) FA and Normative Percentile

The high performance (HP) group (*n* = 21) had a mean RIFO FA of 0.272 (SD = 0.030, SE = 0.007), the low performance (LP) group (*n* = 14) had a mean RIFO FA of 0.266 (SD = 0.028, SE = 0.008), and the moderate performance (MP) group (*n* = 35) had a mean RIFO FA of 0.289 (SD = 0.028, SE = 0.005).

**Figure 5:**
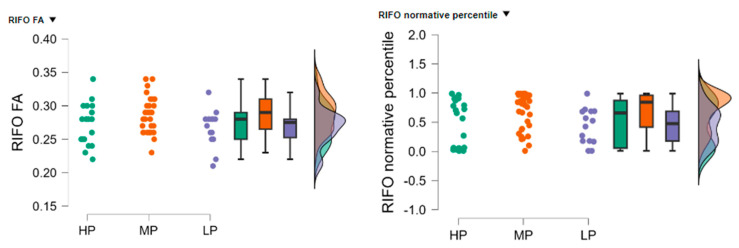
Distribution of RIFO FA (**left**) and normative percentile (**right**) values across the HP, MP, and LP groups.

The HP group (*n* = 21) had a mean normative percentile of 0.492 (SD = 0.399, SE = 0.087), the LP group (*n* = 14) had a mean normative percentile of 0.436 (SD = 0.303, SE = 0.081), and the MP group (*n* = 35) had a mean normative percentile of 0.695 (SD = 0.310, SE = 0.052), see Figure 5.

The Kruskal-Wallis test was conducted to evaluate the differences in RIFO FA and normative percentile values among the three groups. The test indicated a statistically significant difference in RIFO FA values between the groups (H = 7.619, *p* = 0.022) and in RIFO normative percentile values (H = 8.170, *p* = 0.017).

Dunn’s post hoc tests were performed to identify specific group differences. For RIFO FA values, there was a significant difference between the HP and MP groups (z = −2.102, *p* = 0.037) and between the MP and LP groups (z = 2.379, *p* = 0.017). No significant differences were found between the HP and LP groups (z = 0.499, *p* = 0.618).

For RIFO normative percentile values, there was a significant difference between the HP and MP groups (z = −2.093, *p* = 0.039) and between the MP and LP groups (z = 2.525, *p* = 0.012). No significant differences were found between the HP and LP groups (z = 0.640, *p* = 0.522). Dunn’s post hoc tests were performed to identify specific group differences, and the results are summarized in Table 6.

### 3.8. ANCOVA and Covariate Controls

An ANCOVA was conducted with raw RIFO FA as the dependent variable, cognitive group (HP, MP, LP) as a fixed factor, and age (covariate), along with gender (fixed factor) as controls. The analysis revealed a significant main effect of Group, F (2, 65) = 4.30, *p* = 0.018, with a partial η^2^ = 0.12, corresponding to a moderate effect size (Cohen’s f ≈ 0.35). In this model, age significantly predicted raw RIFO FA (*p* < 0.01), whereas gender did not reach significance (*p* > 0.10). These results indicate that group-level differences in RIFO FA persist even after adjusting for demographic factors.

An ANCOVA was also performed using RIFO normative percentiles as the dependent variable, with Group as a fixed factor and Age (covariate) plus Gender (fixed factor) included in the model. The group effect was borderline significant, F (2, 65) = 2.90, *p* = 0.061, with a partial η^2^ ≈ 0.08 and a corresponding Cohen’s f ≈ 0.30. In this model, neither age nor gender significantly predicted RIFO normative percentiles, suggesting that once typical age- and sex-related variations are accounted for, the differences among cognitive groups become more modest.

## 4. Discussion

In this study, our goal was to investigate the relationship between fractional anisotropy (FA) values in several key white matter tracts and cognitive performance across various levels of cognitive impairment. Our findings revealed differences in both raw FA and normative percentile values among the groups; however, the results were not uniformly significant across all comparisons. Notably, when we applied a Bonferroni correction to account for the multiple comparisons performed, some effects that were initially significant became borderline. This conservative correction method effectively minimizes the risk of Type I errors but also increases the likelihood of Type II errors, particularly in an exploratory study with a limited sample size. Consequently, although the overall trends suggest that FA may indeed reflect changes in white matter integrity related to cognitive function, the complex and variable nature of these results indicates that further investigation is needed. Future studies employing larger cohorts and alternative statistical methods, such as false discovery rate (FDR) corrections, may provide a more balanced approach to assessing the clinical utility of FA as a biomarker for cognitive impairment.

Our ANCOVA analyses, controlling for age and gender, provided important insights into group differences in white matter integrity. For raw FA values, the analysis revealed a significant main effect of cognitive group in both the LSLF-A and RIFO tracts, with moderate effect sizes. In these models, age emerged as a significant predictor of raw FA values, while gender did not contribute significantly. By contrast, when FA values were expressed as normative percentiles—which inherently adjust for typical age- and sex-related variations—the group differences were more modest and only approached significance, with effect sizes remaining in the moderate range. This pattern suggests that while raw FA measures capture meaningful group differences, partly driven by age-related changes, the normative percentile approach minimizes the influence of demographic factors and yields more conservative estimates. Overall, these findings imply that the observed differences in white matter integrity between cognitive groups are not solely attributable to age or gender, thereby reinforcing the potential utility of FA as a biomarker for cognitive function. Future research with larger samples would be necessary to further clarify the clinical significance of these measures.

**Figure 6:**
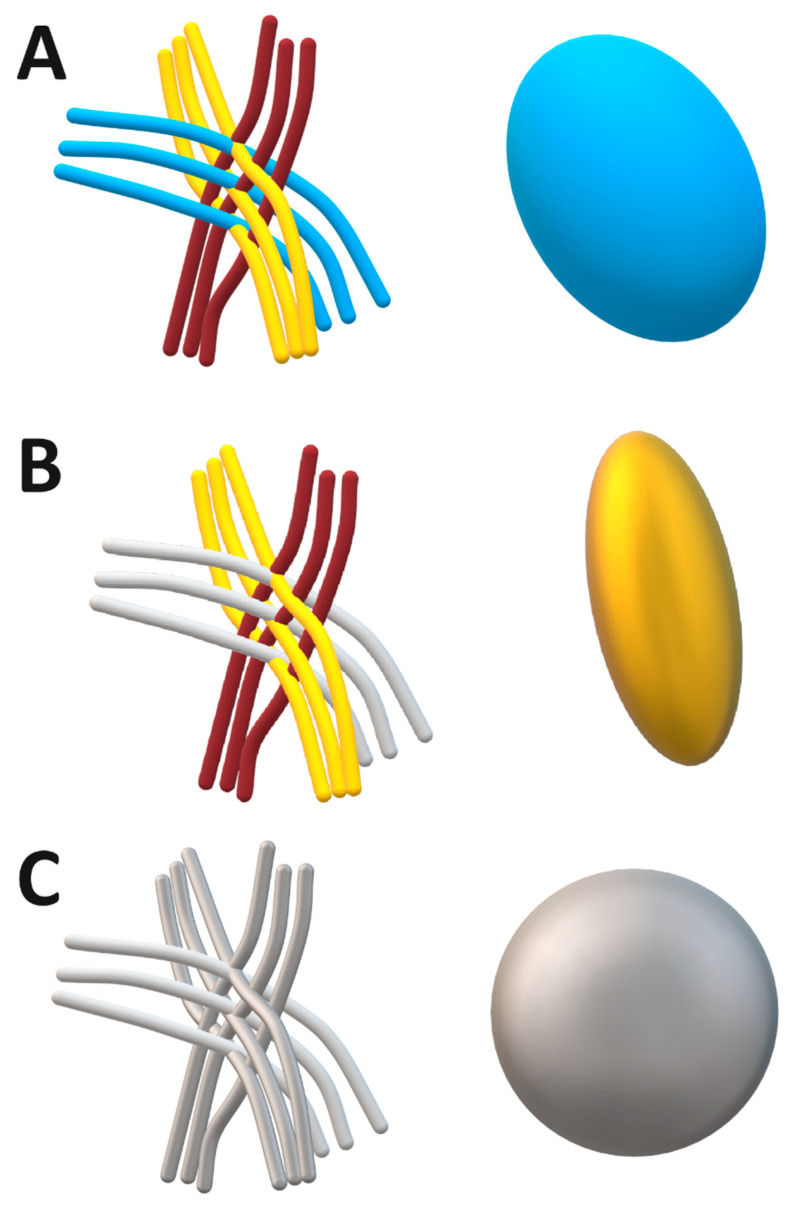
Concept of fractional anisotropy (FA) and its implications for white matter integrity analysis using FA values. (**A**)—three nerve bundles (blue, brown, yellow) representing moderate FA value, where ellipsoid would be elongated, but not as much as in panel (**B**), signifying moderate FA values. This indicates that water molecules predominantly diffuse along the direction of the fibers, but with some degree of diffusion in other directions. (**B**)—two nerve bundles (brown and yellow) intact, grey nerve bundle damaged, resulting in increased isotropic diffusion and higher FA value. (**C**)—all nerve bundles are depicted in grey, indicating significant loss of organization and alignment, low FA value, representing severe white matter damage and disorganization, typically associated with significant white matter damage and advanced neurodegenerative conditions.

***FA changes and complexity of white matter changes in Cognitive Decline.*** The observed variability in fractional anisotropy (FA) across different cognitive impairment levels shows the relationship between white matter integrity and cognitive decline. However, the relationship between FA and cognitive decline is complex, as it is influenced by several factors, including the extent of fiber alignment, the presence of structural damage, and the differential vulnerability of various white matter tracts [27,28].As white matter damage progresses, FA values typically decrease, reflecting a loss of fibre coherence and increased isotropic diffusion. However, in the presence of partially preserved tracts or compensatory mechanisms may sustain higher FA values in certain regions, even in the face of cognitive decline [29,30], see Figure 6.

This complexity highlights the need to consider FA alongside other biomarkers to fully understand white matter changes in cognitive decline.

***Influence of individual variability on fractional anisotropy measurements.*** Individual variability plays a significant role in fractional anisotropy (FA) values, which can complicate the interpretation of FA as a biomarker for cognitive decline and complicate normative value assessment. Factors such as age [10,14,31], genetic predispositions [32], lifestyle [33], and comorbid conditions [34] can all influence white matter integrity and FA values. Additionally, individual differences in brain anatomy, such as the size and orientation of white matter tracts, can lead to variability in FA measurements across participants [35]. This variability may obscure the relationship between FA and cognitive function, making it challenging to draw definitive conclusions. Therefore, it is essential to account for individual differences when interpreting FA data and to consider these factors when developing FA-based biomarkers for cognitive impairment. Further and longitudinal research with larger, more diverse populations is needed to better understand the impact of individual variability on FA and its implications for assessing cognitive decline [36].

***Limitations of segmented white matter tracts.*** The selection of specific white matter tracts for analysis presents certain limitations that could affect the interpretation of our findings. While the tracts chosen in this study, such as the SLF-A, IFO, and cingulum, are relevant to cognitive function, they do not encompass the entirety of the brain’s white matter network [37,38,39]. Cognitive impairment is a complex process that may involve widespread changes across multiple tracts, some of which may not have been captured in our analysis. Additionally, the focus on tracts may overlook the contribution of other regions that are equally important in the progression of cognitive decline. This limited scope could lead to an incomplete understanding of the relationship between fractional anisotropy (FA) and cognitive performance.

***Comparison with previous studies and FA differences in groups.*** We compared our study with selected other studies that investigated fractional anisotropy and cognitive performance. Table 7 summarizes some of the main characteristics of these studies, including sample size, methods, tracts analysed, and key findings.

Our findings support FA as a sensitive marker of white matter integrity related to cognitive function, but they also underscore the limitations of relying on FA alone. Variability across tracts and performance groups suggests that FA must be interpreted within a multimodal framework that includes complementary diffusion metrics and macrostructural measures. Such integration may provide a more comprehensive view of early neurodegenerative changes and improve the reliability of diffusion imaging biomarkers.

Although our sample size restricts the strength of conclusions—particularly for the low-performance group—the observed trends are consistent with evidence that microstructural changes often precede overt clinical decline. Future work with larger, longitudinal, and more diverse cohorts should clarify the role of FA in specific cognitive domains and further assess its value for early detection and risk stratification in cognitive impairment.

## 5. Conclusions

Our study identified significant differences in fractional anisotropy (FA) values and normative percentiles across high, moderate, and low cognitive performance groups, as defined by MoCA scores. These differences were most prominent in the Left and Right Superior Longitudinal Fasciculus—Arcuate (LSLF-A, RSLF-A) and the Left and Right Inferior Fronto-Occipital Fasciculus (LIFO, RIFO) tracts. Intriguingly, the moderate performance group generally exhibited higher FA values compared to the low performance group, suggesting a non-linear relationship between FA and cognitive decline. These findings indicate that FA may serve as a sensitive marker of white matter integrity in relation to cognitive function, but the variability across different tracts underscores the complexity of using FA as a standalone biomarker. The non-linear trend challenges a simple decline model and may reflect compensatory mechanisms or other complex neurobiological processes. While our study provides valuable preliminary evidence, the modest sample size, particularly in the low-performance group, limits definitive conclusions and highlights the need for larger, multimodal studies to validate FA’s role in the early detection and diagnosis of cognitive impairment. Future research should also explore the influence of unmeasured confounders and the relationship between FA and specific cognitive domains to provide a more comprehensive understanding of the underlying mechanisms.

## Figures and Tables

**Table 1:** Descriptive statistics for each group: High Performance (HP), Moderate Performance (MP), and Low Performance (LP).

Statistic	Age (HP)	Age (MP)	Age (LP)	MoCA (HP)	MoCA (MP)	MoCA (LP)
N	21	35	14	21	35	14
Participants (M:F)	1:20	7:28	5:9	1:20	7:28	5:9
Median	72.0	74.0	79.5	27.0	23.0	10.0
Mean	72.6	72.7	75.9	27.3	22.7	9.7
Std. Error of Mean	1.1	1.1	3.0	0.3	0.4	1.2
Std. Deviation	5.2	6.8	11.1	1.1	2.3	4.4
Minimum	61.0	57.0	62.0	26.0	18.0	4.0
Maximum	83.0	85.0	96.0	30.0	25.0	16.0

**Table 2:** Descriptive statistics for each white matter tract in group (high performance (HP), moderate performance (MP), low performance (LP), Left Corticospinal Tract (LCST FA, LCST normative percentile), Right Corticospinal Tract (RCST FA, RCST normative percentile), Left Superior Longitudinal Fasciculus—Arcuate (LSLF-A FA, LSLF-A normative percentile), Right Superior Longitudinal Fasciculus—Arcuate (RSLF-A FA, RSLF-A normative percentile), Left Inferior Fronto-Occipital Fasciculus (LIFO FA, LIFO normative percentile), Right Inferior Fronto-Occipital Fasciculus (RIFO FA, RIFO normative percentile), Left Cingulum (LCin FA, LCin normative percentile), Right Cingulum (RCin FA, Rcin normative percentile)).

		Mean	Std. Deviation	Minimum	Maximum
LCST FA	HP	0.443	0.047	0.370	0.550
	MP	0.459	0.042	0.390	0.580
	LP	0.458	0.037	0.400	0.540
LCST normative percentile	HP	0.669	0.314	0.010	0.990
	MP	0.767	0.215	0.181	0.990
	LP	0.789	0.212	0.208	0.990
RCST FA	HP	0.435	0.051	0.350	0.530
	MP	0.452	0.038	0.390	0.550
	LP	0.449	0.037	0.380	0.510
RCST normative percentile	HP	0.632	0.348	0.010	0.990
	MP	0.798	0.169	0.228	0.990
	LP	0.785	0.201	0.341	0.976
LSLF-A FA	HP	0.310	0.035	0.240	0.370
	MP	0.329	0.031	0.270	0.390
	LP	0.306	0.024	0.260	0.330
LSLF-A normative percentile	HP	0.536	0.392	0.010	0.990
	MP	0.731	0.281	0.095	0.990
	LP	0.524	0.347	0.028	0.942
RSLF-A FA	HP	0.313	0.036	0.260	0.390
	MP	0.329	0.022	0.290	0.370
	LP	0.306	0.021	0.270	0.340
RSLF-A normative percentile	HP	0.526	0.383	0.010	0.990
	MP	0.778	0.198	0.229	0.990
	LP	0.514	0.299	0.093	0.956
LIFO FA	HP	0.289	0.034	0.240	0.360
	MP	0.308	0.032	0.240	0.390
	LP	0.283	0.029	0.230	0.340
LIFO normative percentile	HP	0.487	0.386	0.010	0.990
	MP	0.738	0.274	0.010	0.990
	LP	0.484	0.329	0.010	0.990
RIFO FA	HP	0.272	0.030	0.220	0.340
	MP	0.289	0.028	0.230	0.340
	LP	0.266	0.028	0.210	0.320
RIFO normative percentile	HP	0.492	0.399	0.010	0.990
	MP	0.695	0.310	0.010	0.990
	LP	0.436	0.303	0.010	0.990
LCin FA	HP	0.316	0.035	0.260	0.400
	MP	0.329	0.026	0.270	0.370
	LP	0.319	0.029	0.260	0.350
LCin normative percentile	HP	0.527	0.384	0.010	0.990
	MP	0.738	0.265	0.010	0.990
	LP	0.644	0.326	0.017	0.973
RCin FA	HP	0.313	0.036	0.250	0.380
	MP	0.326	0.026	0.280	0.370
	LP	0.311	0.029	0.250	0.350
Rcin normative percentile	HP	0.569	0.363	0.010	0.990
	MP	0.765	0.241	0.075	0.990
	LP	0.617	0.294	0.027	0.980

**Table 3:** LSLF-A FA and normative percentile Dunn Post hoc test results comparing cognitive performance groups (Z—Dunn’s post hoc test statistic; Wi, Wj = rank sums for each comparison group; *p* = unadjusted *p*-value; *p*^bonferroni^—Bonferroni-corrected *p*-value).

Comparison		Z	Wi	Wj	*p*	*p* ^bonferoni^
FA values	HP–MP	−1.922	30.714	41.457	0.055	0.164
	HP–LP	0.419	30.714	27.786	0.675	1.000
	MP–LP	2.135	41.457	27.786	0.033	0.098
FA normative percentiles	HP–MP	−1.957	30.548	41.529	0.050	0.151
	HP–LP	0.384	30.548	27.857	0.701	1.000
	MP–LP	2.127	41.529	27.857	0.033	0.100

**Table 4:** RSLF-A FA and normative percentile Dunn Post hoc test results comparing cognitive performance groups (Z—Dunn’s post hoc test statistic; Wi, Wj = rank sums for each comparison group; *p* = unadjusted *p*-value; *p*^bonferroni^—Bonferroni-corrected *p*-value).

Comparison		Z	Wi	Wj	*p*	*p* ^bonferoni^
FA values	HP–MP	−2.179	30.333	42.486	0.088	0.059
	HP–LP	0.652	30.333	25.786	0.514	1.000
	MP–LP	2.614	42.486	25.786	0.009	0.027
FA normative percentiles	HP–MP	−2.124	30.548	42.329	0.034	0.101
	HP–LP	0.617	30.548	26.071	0.537	1.000
	MP–LP	2.528	42.329	26.071	0.011	0.034

**Table 5:** LIFO FA and normative percentile Dunn Post hoc test results comparing cognitive performance groups (Z—Dunn’s post hoc test statistic; Wi, Wj = rank sums for each comparison group; *p* = unadjusted *p*-value; *p*^bonferroni^—Bonferroni-corrected *p*-value).

Comparison		Z	Wi	Wj	*p*	*p* ^bonferoni^
FA values	HP–MP	−2.209	29.833	42.157	0.027	0.082
	HP–LP	0.355	29.833	27.357	0.723	1.000
	MP–LP	2.315	42.157	27.357	0.021	0.062
FA normative percentiles	HP–MP	−2.528	28.667	42.843	0.011	0.034
	HP–LP	0.182	28.667	27.393	0.856	1.000
	MP–LP	2.404	42.843	27.393	0.016	0.049

**Table 6:** RIFO FA and normative percentile Dunn Post hoc test results comparing cognitive performance groups (Z—Dunn’s post hoc test statistic; Wi, Wj = rank sums for each comparison group; *p* = unadjusted *p*-value; *p*^bonferroni^—Bonferroni-corrected *p*-value).

Comparison		Z	Wi	Wj	*p*	*p* ^bonferoni^
FA values	HP–MP	−2.102	30.333	42.057	0.037	0.107
	HP–LP	0.499	30.333	26.857	0.618	1.000
	MP–LP	2.379	42.057	26.857	0.017	0.052
FA normative percentiles	HP–MP	−2.093	30.524	42.271	0.039	0.109
	HP–LP	0.640	30.524	26.036	0.522	1.000
	MP–LP	2.525	42.271	26.036	0.012	0.035

**Table 7:** Comparison of our study with representative prior work in the field of white-matter integrity and cognition.

Study (Author, Year)	Sample Size	Methods/Focus	Tracts Analyzed	Key Findings/Distinction
Ezzati et al., 2016 [40]	100	DTI with probabilistic tractography; examined hippocampal volume and cingulum FA; linear regression relating imaging measures to verbal memory	Left cingulum, hippocampal volume	Lower left hippocampal volume and decreased FA in the left cingulum were independently associated with poorer verbal episodic memory.
Lancaster et al., 2016 [41]	51	Baseline DTI processed via tract-based spatial statistics; diffusion metrics (FA and axial diffusivity) in the medial temporal	Cingulate-hippocampal tract, fornix/stria terminalis, uncinate fasciculus	Greater axial diffusivity and lower FA in the cingulate-hippocampal and uncinate tracts predicted greater decline in delayed recall over three years.
Grieve et al., 2007 [10]	87	DTI cross-sectional voxel-based and ROI analysis; examined relationships between age, FA, and executive function	Frontal, parietal temporal lobes; prefrontal-parietal networks	FA declines with age; negative association with executive function; prefrontal-parietal involvement
Peter et al., 2025 [19]	4467 (9208 sessions across 9 cohorts)	Large-scale multisite dMRI; FW and FW-corrected metrics (FA, MD, AD, RD) across 48 tracts; longitudinal ComBat harmonization; assessed associations with cognitive composites (memory, executive function, language) and AD biomarkers (amyloid, tau, APOE ε4, hippocampal volume).	48 tracts, including limbic (cingulum, fornix), association, projection, and transcallosal tracts.	FW showed the strongest and most consistent associations with cognitive decline, especially in memory. Limbic tracts (cingulum, fornix) were most vulnerable. FW interacted with APOE ε4 status, hippocampal volume, and amyloid positivity to predict accelerated decline.
Sun et al., 2025 [17]	ADNI: 328; GHABS: 78 (cross-sectional + longitudinal data, multiple visits)	Multimodal imaging (dMRI/DTI, T2-FLAIR, Aβ PET, tau PET); investigated sequential associations of microstructural (FA) and macrostructural (WMH) degeneration with age, vascular risk, and AD pathology across biological A/T stages.	Cingulum–hippocampus (CGH), fornix (FX), fornix/stria terminalis (FXST), cingulum–cingulate gyrus (CGC), and whole-brain WM regions.	Lower FA levels and faster declines are strongly associated with tau (not Aβ); tau-related FA decreases predicted higher burden and faster progression of WMHs. Microstructural degeneration consistently preceded macrostructural WM changes, and FA mediated both Aβ-related and age-related WMH burden.

## Data Availability

The data that support the findings in this study are available from the corresponding author upon reasonable request.
